# Catalytic Steam-Assisted Pyrolysis of PET for the Upgrading of TPA

**DOI:** 10.3390/ma16062362

**Published:** 2023-03-15

**Authors:** Kuntong Song, Yi Li, Ruiqi Zhang, Nan Wang, Junhong Liu, Wenxia Hou, Qing Zhou, Xingmei Lu

**Affiliations:** 1 Beijing Key Laboratory of Ionic Liquids Clean Process, CAS Key Laboratory of Green Process and Engineering, State Key Laboratory of Multiphase Complex Systems, Institute of Process Engineering, Chinese Academy of Sciences, Beijing 100190, China; 2 Sino Danish College, University of Chinese Academy of Sciences, Beijing 100049, China; 3 School of Chemical Engineering, University of Chinese Academy of Sciences, Beijing 100049, China; 4 Henan Institute of Advanced Technology, Zhengzhou University, Zhengzhou 450001, China

**Keywords:** PET, metal–acid catalyst, steam-assisted pyrolysis, TPA

## Abstract

Compared with conventional pyrolysis, steam-assisted pyrolysis of polyethylene terephthalate (PET) can effectively eliminate char and upgrade terephthalic acid (TPA). However, during steam-assisted pyrolysis of PET, the degree of cracking still varies greatly, and while some of the product is excessively cracked to gas, the other part is still insufficiently cracked. In addition, these two types of products seriously affect the yield and purity of TPA. To further enhance the TPA, an attempt was made to reduce these impurities simultaneously by synergistic catalysis among the different components of the metal–acid catalyst. Through a series of experiments, Pt@Hzsm-5 was screened as the optimal catalyst. In the catalytic steam-assisted pyrolysis of PET, the optimum reaction temperature decreased to 400 °C, the calculated yield of TPA increased to 98.23 wt%, and the purity increased to 92.2%. The Pt@Hzsm-5 could be recycled three times with no significant decrease in the obtained yield of TPA. The catalytic mechanism of the Pt@Hzsm-5 was investigated through the analysis of the products and isotope tracing experiments. The Pt catalyzed the hydrogen transfer reaction between the water molecules and PET molecules, which inhibited the excessive cracking of TPA by improving the hydrogen transfer efficiency, reduced the generation of gaseous products, and improved the calculated yield of TPA. In contrast, the Hzsm-5 catalyzed the reaction of monovinyl ester cracking to TPA, effectively reducing the impurities in the solid product, increasing the olefin yield, and improving the purity of TPA. This discovery not only clarifies the synergistic catalytic effect of the Pt@Hzsm-5 in the steam-assisted pyrolysis of the PET reaction but also lays the foundation for further screening of other inexpensive metal–acid catalysts. This is of great significance to realize the industrial application of TPA preparation by PET pyrolysis.

## 1. Introduction

As an important petrochemical product, the global apparent usage of PET reached 78 million tons in 2019 [1]. Due to the stability of PET, it is difficult to degrade spontaneously in the natural environment, so these waste plastics not only waste a large amount of nonrenewable oil resources but also pollute the environment [2,3,4]. Therefore, the realization of the efficient recycling of waste PET is of great significance both in terms of environmental protection and resource conservation.

The methods currently used to achieve PET recycling are thermoplasticity [5] and degradation [6]. Thermoplasticity has better economy and is currently the most common method to achieve the recycling of waste PET. However, the quality of the product obtained by this method appears to be degraded and is not a true closed-loop cycle. In contrast to the unsustainability of thermoplasticity, degradation is a reliable way to achieve the closed-loop recycling of waste PET. There are many studies on the degradation of PET, including bioremediation [7,8], glycolysis [9,10], aminolysis [11,12], and hydrolysis [13,14]. However, with these degradation methods, issues regarding product quality or production costs cannot be effectively addressed, which makes them difficult to be scaled up. Correspondingly, pyrolysis, as a simple and efficient technology, is considered to be a reliable method for recycling waste PET, and it has obvious potential for industrialization [15,16,17,18]. However, there are still some problems with the pyrolysis of PET. Studies have shown that the process of pyrolysis of PET not only generates greenhouse gases such as CO_2_ but also forms a large amount of unavoidable char [19,20,21]. Maite Artetxe et al. [20] investigated the pyrolysis process of PET using rapid pyrolysis in a sprayed bed reactor. The results showed that the solid organic products obtained were very complex, with more than 30 species in the temperature range of 500 °C–600 °C pyrolysis. The highest yield of benzoic acid could be 26.98%, and terephthalic acid was not detected. In addition, the product contained 7% char, which was not separable from these organic products as a solid powder, encapsulated on the surface of quartz sand. These byproducts, due to the fact of excessive pyrolysis, not only reduce the yield of the target product TPA but also cause environmental pollution and increase the difficulty of separation.

In a previous study, it was found that the production of char could effectively be inhibited under a steam atmosphere, and the yield of TPA could be improved [22]. Through the study, it was found that as an exogenous hydrogen source, water effectively inhibited the excessive cracking of PET during the pyrolysis through an intermolecular hydrogen transfer reaction. By optimizing the reaction conditions, a TPA product with a 72.5% yield and 85.5% purity was obtained at 450 °C. It was found that although the excessive cracking of the TPA during pyrolysis was effectively inhibited by the steam, there were still some problems that needed to be solved.

It was noticed that during the steam-assisted pyrolysis of PET, although no char was produced, there was still some TPA, which was further cracked to carbon dioxide and other gases [23]. Moreover, some impurities that were not adequately cracked appeared due to the inhibitory effect of the steam on the cracking reaction of the PET. It was found that the main impurity in the TPA obtained by steam-assisted pyrolysis of PET was mono-terephthalate (mTPA), which is an intermediate product that requires further cracking to obtain TPA. In fact, both gaseous products and solid impurities (such as mTPA) have an impact on the yield and purity of the TPA product. Therefore, to further upgrade TPA, it was not only necessary to improve the efficiency of the intermolecular hydrogen transfer to inhibit the excessive cracking of TPA but also to promote the continued cracking of mTPA at low temperatures. Therefore, in order to achieve the goal of upgrading TPA, it is necessary to achieve precise control of the reaction.

In order to achieve this goal, a suitable catalyst is essential. However, at present, there are relatively few studies on the catalytic pyrolysis of PET. Kratish et al., at Northwestern University, USA, using 1.2-ethyleneglycol dibenzoate as a model compound for PET and heterooxygen atoms of Mo as catalysts for pyrolysis under a hydrogen atmosphere at 260 °C, showed that the secondary cracking of the pyrolysis gas could be effectively suppressed, and ethylene-rich pyrolysis gas could be obtained. Toshiaki Yoshioka and other researchers reported an industrial yield of 22% terephthalic acid in pyrolysis experiments and further used CaO as a decarboxylation catalyst to achieve PET-catalyzed decarboxylation to benzene-rich oil in a filled-tube reactor at 700 °C, indicating that the strength of the alkalinity of calcium oxide is a key influencing factor for decarboxylation, and the strong alkalinity of CaO is beneficial for improving the selectivity of aromatic hydrocarbons. On the one hand, the main goal of these catalytic pyrolysis studies was not to achieve the efficiency and quality enhancement of TPA products and, on the other hand, these catalytic pyrolysis reactions were also mainly focused on conventional pyrolysis reactions. No studies have been reported on the catalytic enhancement of TPA in steam-assisted pyrolysis systems.

For the purpose of catalytic hydrogenation, the active component of the catalyst is determined. It was noticed that noble metals, such as platinum (Pt) [24,25,26] and palladium (Pd) [27,28,29], are the most widely used metal components in industrial hydrogenation catalysts due to the fact of their good hydrogenation activity and excellent stability. However, their hydrogenation ability in the PET steam-assisted pyrolysis process has not been explored. Therefore, Pt and Pd were selected as the active components and preferred in this study. Meanwhile, it was also noticed that some Bronsted acids, such as Hzsm-5, ZSM-5, SBA-15, and Al_2_O_3_, are effective cracking catalysts in petroleum refining processes and pyrolysis recovery of waste plastics [30,31] (such as PE) due to the catalytic effect of acidic sites [32,33,34]. Therefore, combining them to prepare and screen a metal–acid bifunctional catalyst with noble metal as the active center and Bronsted acid as the carrier to utilize their synergistic catalytic ability in order to simultaneously achieve the two purposes of inhibiting the excessive cracking of TPA and catalyzing the continued cracking of mTPA was considered.

The aim of this study was to (1) find an efficient metal–acid catalyst for upgrading TPA products during the steam-assisted pyrolysis of PET; (2) determine the optimal catalytic conditions for steam-assisted pyrolysis and the highest values for TPA yield and purity by optimizing the reaction conditions; (3) analyze the solid- and gas-phase products using in situ gas-phase analysis techniques and isotope tracking; and (4) investigate the role of each component of the catalyst in the reaction process.

## 2. Materials and Methods

The equipment used for the experiments was a fixed-bed reactor with a steam preheater, as shown in Figure S1 and detailed in the Supplementary Materials. The PET used for the experiments was purchased from DuPont, and it was crushed to less than 0.178 mm before use, and the mass of PET taken for each experiment was 2.0 g. The corresponding mass of catalyst was evenly spread on a sieve plate inside a quartz tube. Before the start of the experiment, the reactor was purged with nitrogen to replace the residual air at a flow rate of 200 mL/min, which was set to 20 mL/min during the experiment. In addition, it was constant in all experiments. The reaction temperature interval was set in the range of 350–600 °C in steps of 50 °C. The heating rate was set to 0.5 °C/min, 1.0 °C/min, 2.5 °C/min, 5.0 °C/min, and 10.0 °C/min, and the water flow rate was set to 0.05 mL/min, 0.1 mL/min, 0.2 mL/min, 0.5 mL/min, and 1.0 mL/min. The masses of the catalysts were 0.05 g, 0.1 g, 0.2 g, 0.4 g, and 0.6 g, and the loading metal mass ratios were 0.5 wt%, 1.0 wt%, and 2.0 wt%, respectively.

To ensure the precise control of the reaction conditions, sophisticated temperature control instruments were used. The water flow rate during the reaction was controlled by microcirculation, using a microcirculation pump to deliver water to the steam preheater and heat it to 140 °C to produce steam, which was then mixed thoroughly with nitrogen in the pipeline. The flow rate of the nitrogen was constant at 20 mL/min in all reactions, which was much lower than the steam flow rate and only served as a carrier gas. Before the start of the pyrolysis reaction, the PET powder was taken into a quartz basket and suspended from a wire in the middle of the fixed-bed reactor. A removable quartz tube liner was placed on the inner wall of the fixed pyrolyzer in order to avoid the catalytic effect of the metallic tube wall on the reaction and, thus, interfering with the experimental results.

Chloroplatinic acid and palladium chloride, purchased from Aladdin, were used as the metal precursors for platinum and palladium, respectively. The Hzsm-5, ZSM-5, SBA-15, and Al_2_O_3_, purchased from Aladdin, were used as carriers. All catalysts were synthesized by the sol-gel method [35]. The precursor containing 100 mg of metal was mixed with 200 mg of polyvinyl alcohol (PVA) solution and stirred in a beaker at room temperature for 60 min. The corresponding proportion of carrier powder (all ground to below 0.178 mm) was added and stirred for 120 min. A total of 0.1 mol/L of sodium borohydride (NaBH_4_) solution was added dropwise and stirred for 180 min. The solid sample obtained by filtration was repeatedly washed with deionized water to remove the residual chloride ions and placed in an oven at 70 °C for 24 h. The dried sample was removed and placed in a muffle furnace, heated from room temperature to 750 °C at a heating rate of 2 °C/min, and held for 300 min. After calcination, the samples were removed and placed in a horizontal tube furnace, purged with hydrogen gas for 30 min, and then heated from room temperature to 750 °C at a heating rate of 2 °C/min and held for 120 min. In addition, the final obtained products were named Pt (Pd)@Hzsm-5, -ZSM5, -SBA-15, and -Al_2_O_3_, respectively. The analytical equipment used in this study, such as the SEM (JSM 7001F, Hitachi, Tokyo, Japan), TEM (JEM-2100F, Hitachi, Tokyo, Japan), XPS (AXIS SUPRA+, Shimadzu, London, UK), TG (DTG-60H, Shimadzu, Tokyo, Japan), HPLC (LC-20AT, Shimadzu, Tokyo, Japan), GC-TCD (Micro-490, Angilent, LA, USA), ESI-MS (QTOF, Bruker, Berlin, Germany), and the analytical methods for the products and catalysts were the same as in previous studies [36].

Due to the involvement of water in the reaction process, a portion of the atoms in the obtained TPA are not entirely derived from the PET. Therefore, the mass ratio of TPA to PET alone cannot describe the formation of TPA with complete accuracy. At this point, it is noticed that since the benzene ring in the TPA comes entirely from PET, the mass of this fraction is not disturbed by water. Therefore, it was defined that the mass ratio between the product and the benzene ring in the raw material was used to calculate the yield to reflect the production of TPA as follows [22]. Among them, mTPAbr is the mass of the benzene ring in the produced TPA, and mPETbr is the mass of the benzene ring in the initially added PET.

(1) $$\;YTPA\left(\%\right)=\frac{m_{TPAbr}}{m_{PETbr}}*100\%$$

To ensure the accuracy of the data, all experiments were performed at least three times in parallel. The experimental groups under different conditions were subjected to error bar analysis. The error analysis determined that the errors obtained from the experiments were all within 5%, thus confirming that the data in the study are reliable.

## 3. Results and Discussion

### 3.1. Effect of the Reaction Path during PET Pyrolysis

In a previous study [22], it was noticed that water can contribute to enhancing the yield of TPA through both the hydroxyl attack pathway and hydrogen transfer pathway. To further clarify the effect of water on the different reaction pathways, isotope experiments using deuterated water were conducted as a tracer reagent. The deuterated water was used instead of water in the reaction, and the resulting products were analyzed by ESI-MS.

**Figure 1:**
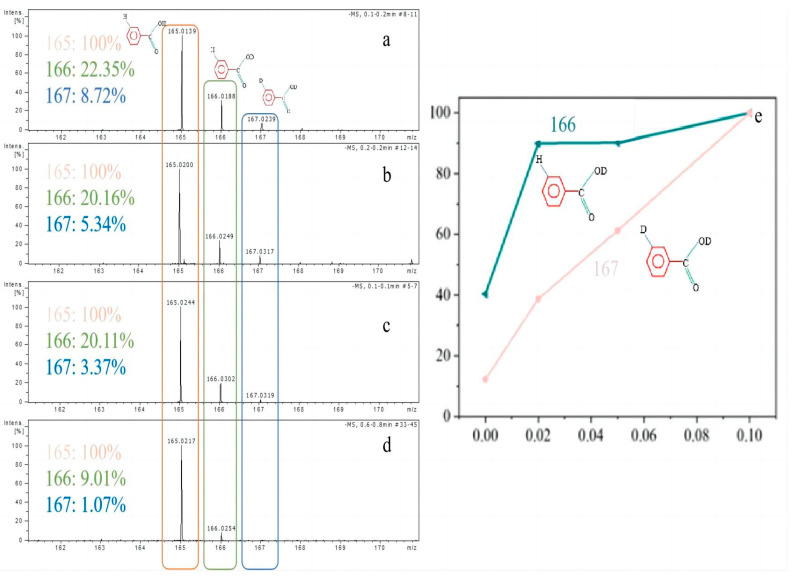
ESI–MS analysis of the solid products. (**a**) Flow rate (D_2_O): 0.1 mL/min; (**b**) flow rate (D2O): 0.05 mL/min; (**c**) flow rate (D_2_O): 0.02 mL/min; (**d**) flow rate (H_2_O): 0.1 mL/min; (**e**) variation of the relative content of molecules with an m/z of 166 and 167 for the different D_2_O flow rates.

The results are shown in Figure 1. When the water flow velocity was lower than 0.1 mL/min, the relative content of molecules with a mass-to-charge ratio (m/z) of 167 decreased rapidly from 8.72% to 3.37% as the flow rate decreased, but the change in the relative content of molecules with an m/z mass ratio of 166 was not significant. In previous studies [22], it has been clarified that a molecule with an m/z of 166 represents a reaction in which a hydroxyl group attacked the ester bond, and a molecule with an m/z of 167 represents a hydrogen transfer reaction on the benzene ring in addition to the hydroxyl group attack. As shown in Figure 1e, when the water flow rate was 0.02 mL/min, the hydroxyl attack reaction had fully occurred, but the hydrogen transfer reaction on the benzene ring was incompletely carried out.

The decrease in the number of molecules with an m/z of 167 means that fewer hydrogen transfer reactions occurred on the benzene ring, more coke was produced, and the yield and purity of the TPA decreased. Therefore, it can be determined that the hydrogen transfer reaction on the benzene ring occurred with more difficulty, and this process should also be mainly considered when screening catalysts.

### 3.2. Selection of Catalysts

**Figure 2:**
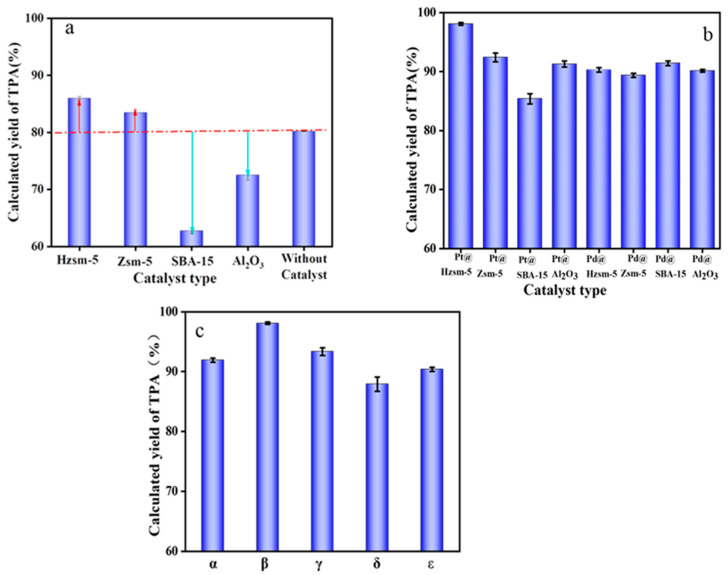
Calculated yields of TPA under different catalytic conditions (temperature: 400 °C; heating rate: 0.5 °C/min; flow rate: 0.1 mL/min): (**a**) catalytic effect of various carriers; (The red arrow means that the yield increases, and the blue arrow means that the yield decreases) (**b**) catalytic effect of Pt and Pd catalysts with different carriers (metal loading—1 wt%; catalyst to PET feeding ratio—1:10); (**c**) effect of Pt loading and the feeding ratio on the catalytic effect of the Pt@Hzsm-5 (α Pt loading—0.5 wt%; feeding ratio—1:10; β, Pt loading—1.0 wt%; feeding ratio—1:10; γ, Pt loading—2.0 wt%; feeding ratio—1:10; δ, Pt loading—1.0 wt%; feeding ratio—1:20; ε, Pt loading—1.0 wt%, feeding ratio—1:5).

As shown in Figure 2a,b, the catalytic effects of various carriers and catalysts were investigated in order to screen the optimal catalyst. To control the variables, in these experiments, the reaction temperature was always 400 °C, the heating rate was 0.5 °C/min, the water flow rate was 0.1 mL/min, the feeding ratio of catalyst to PET was 1:10, and the metal loading of both Pt and Pd was 1 wt%. The results showed that the calculated yield of the TPA obtained under uncatalyzed conditions was 80.23 wt%. As shown in Figure 2a, the Hzsm-5 and Zsm-5 had a positive effect on improving the yield of TPA, but the yield of TPA obtained under the catalysis of the SBA-15 and Al_2_O_3_ was even lower than that of the TPA under uncatalyzed conditions. The order of the catalytic activity of the four carriers is Hzsm-5 > Zsm-5 > Al_2_O_3_ > SBA-15.

The surface acidity and pore size distribution of the carriers were investigated, and the results are shown in Figure S2. It was found that the pore widths of the Hzsm-5 and Zsm-5 were approximately 0.9 nm, while those of the SBA-15 and Al_2_O_3_ were both larger than 6 nm. This implies that the SBA-15 and Al_2_O_3_ were less selective for the size of the molecules, and large molecules could also enter the pores. Therefore, their corresponding solid yields were also relatively low. It was also noticed that the higher the acid content, the higher the solid yield when the pore size distribution was approximate. It was found that the catalytic effect of the carrier was determined by both the pore structure and the acidity, and a smaller pore size and a higher acid content within a certain range were favorable for the generation of TPA solid products.

However, when these carriers were loaded with metals, the catalytic effect was significantly improved in all cases. As shown in Figure 2b, the Pt-based catalysts were relatively effective compared to the Pd-based catalysts. In particular, the calculated yield of the TPA increased to 98.26 wt% when Pt@Hzsm-5 was used as the catalyst. Therefore, Pt@Hzsm-5 was screened as the required catalyst for the reaction. This finding indicates that Pt has a better catalytic activity in the reaction of steam-assisted pyrolysis of PET for the preparation of TPA. Soosan et al. found that Pt@C has a better catalytic effect than Pd@C in conventional PET pyrolysis [37] for the inhibition of harmful substances such as biphenyl generated due to the fact of excessive cracking. This result is consistent with the finding that platinum also better inhibits the excessive cracking of TPA in a steam-assisted pyrolysis reaction.

As shown in Figure 2c, in order to further investigate the effects of the catalyst and PET feeding ratios and metal loading on the reaction, a controlled variable experimental investigation was conducted on them separately. The results showed that the most optimized feeding ratio was 1:10 and the optimal loading was 1 wt%. It was also noticed in Figure 2c that the calculated yield of the TPA decreased more severely in the δ and ε conditions than in the α and γ conditions compared to the optimal condition β, which indicates that the variation of the feeding ratio had a greater effect on the reaction than the variation of the metal loading. This is mainly because the reaction of the catalytic vapor-assisted pyrolysis of PET is a nonhomogeneous gas–solid reaction, and a variation in the catalyst amount can lead to a large change in the gas–solid reaction interface, which has an impact on the calculated yield of TPA.

From these studies, it was concluded that the catalyst carrier is also catalytic for the reaction itself, and that Hzsm-5 has the best effect. Moreover, it was found that increasing the metal loading and catalyst dosage within a certain range is beneficial for increasing the yield of TPA, but as they continue to increase, the yield of TPA decreases. This is mainly due to the fact that an excess of catalyst leads to an overly vigorous reaction, which in turn reduces the yield of solid products. Through a series of controlled experiments, the optimal catalyst was finally screened as Pt@Hzsm-5 with a metal loading of 1 wt% and a feeding ratio of 1:10.

### 3.3. Effect of the Reaction Conditions on the Products

**Figure 3:**
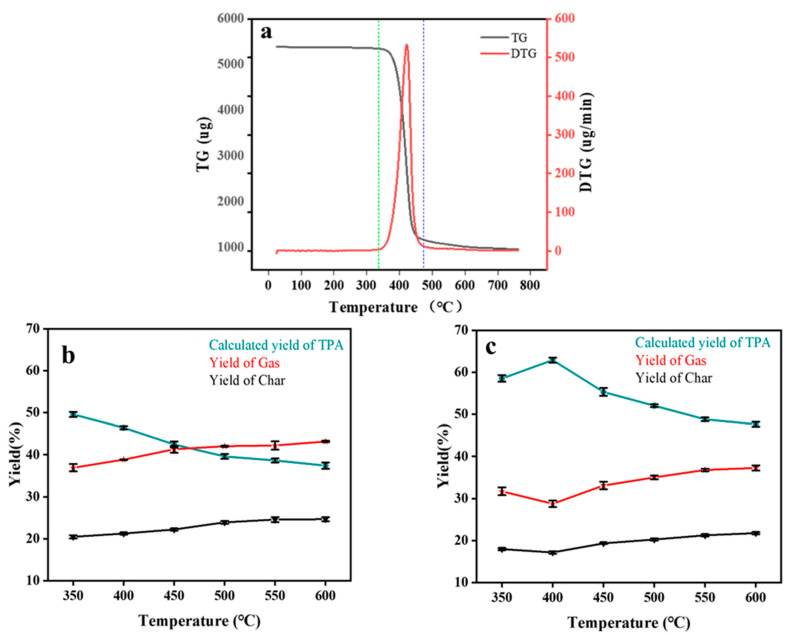
Analysis of PET catalytic conventional pyrolysis products (catalyst—Pt@Hzsm-5; feeding ratio—1:10): (**a**) thermogravimetric analysis (The green line is the initial pyrolysis temperature, and the blue line is the complete pyrolysis temperature); (**b**) product distribution of catalytic pyrolysis at different temperatures; (**c**) product distribution of the catalytic reforming at different temperatures.

As shown in Figure 3a, thermogravimetric experiments were performed to investigate the thermal stability of PET under the catalytic effect of Pt@Hzsm-5. The sample for the thermogravimetric experiments was a mixture of PET and catalyst with a mass ratio of 10:1. The experimental results showed that the reaction temperature interval was 320 °C–480 °C, and there was still approximately 20 wt% residual at 750 °C. In these residues, there was a part of char in addition to the catalyst.

According to the temperature interval of the reaction, the product distribution pattern in the range of 350 °C–600 °C was investigated. As shown in Figure 3b, the calculated yield of TPA was only 50 wt% under the condition that the PET was mixed with the catalyst before pyrolysis, and it decreased with the increase in the temperature. It was also noticed that there was always approximately 20 wt% char present, even rising at high temperatures. In contrast, the calculated yield of the TPA increased when the catalyst was placed on a sieve plate below the PET sample. As shown in Figure 3c, the calculated yield of the TPA was higher than 60 wt% in this reaction of catalytic reforming. In addition, the optimal reaction temperature decreased from 500 °C to 400 °C compared to conventional PET pyrolysis under uncatalyzed conditions. This finding demonstrates that catalytic reforming with a separate arrangement of catalyst and PET is a more efficient technological approach in the pyrolysis reaction system of PET. This is due to the fact that in such a reaction the catalyst and the gaseous reactants are able to have a more adequate contact [38].

### 3.4. Effect of the Reaction Conditions on the Products

**Figure 4:**
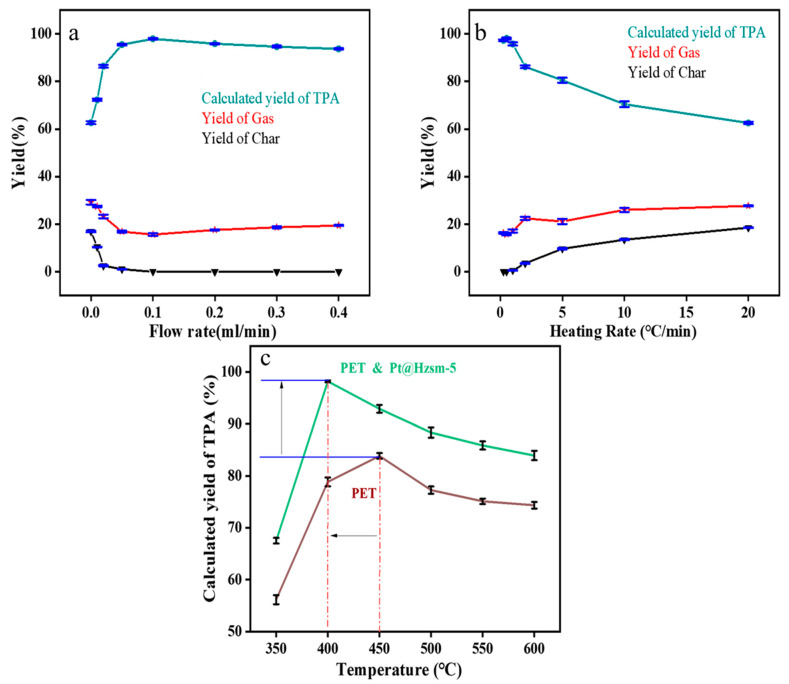
Optimization of the reaction conditions: (**a**) effect of the flow rate on the reaction products; (**b**) effect of the water heating rate on the reaction products; (**c**) effect of the temperature on the reaction products (The red line is the optimal temperature of the two groups of reactions).

As shown in Figure 3, the effects of the water flow rate, heating rate, and temperature on the reaction products were investigated separately to further optimize the reaction conditions and determine the minimum excess vapor coefficient required for the reaction. As shown in Figure 4a, when the water flow rate was less than 0.1 mL/min, the yield of char decreased from 20 wt% to 0 wt% with the increase in the flow rate, while the calculated yield of TPA increased accordingly. In addition, when the water flow rate was greater than 0.1 mL/min, the calculated yield of TPA decreased slightly as the water flow rate increased. This is mainly due to the fact that too large a water velocity would cause a partial loss of the collected TPA.

The effect of the heating rate was also investigated. As shown in Figure 4b, the experimental results showed that the calculated yield of TPA gradually decreased, and the yield of char gradually increased with the increase in the heating rate when the heating rate exceeded 0.5 °C/min under the premise of a water flow rate of 0.1 mL/min. However, the calculated yield of TPA did not change with the heating rate when the heating rate was lower than 0.5 °C/min.

The effect of the pyrolysis temperature was investigated based on the optimized water flow rate and heating rate. As shown in Figure 4c, in the reaction of the catalytic steam-assisted pyrolysis of PET, the calculated yield of TPA showed a rising and then decreasing trend in the range of 350 °C–600 °C and reached a peak of 98.26 wt% at 400 °C. Compared with the experimental results of the uncatalyzed steam-assisted pyrolysis of PET under the same conditions, the Pt@Hzsm-5 catalyst not only effectively improved the calculated yield of TPA but also reduced the optimal reaction temperature from 450 °C to 400 °C. In the catalytic steam-assisted pyrolysis of PET, although its water flow rate and heating rate were the same as those in the uncatalyzed water-assisted pyrolysis of PET, its minimum excess vapor coefficient α also decreased from 150 to 110, because the reaction temperature interval decreased from 130 °C to 80 °C, effectively reducing the water consumption of the reaction and lowering the energy consumption.

Through these studies, the reaction conditions were optimized. At a temperature of 400 °C, a ramp-up rate of 0.5 °C/min and a water flow rate of 0.1 mL/minshi, a TPA product with a yield of 98.26 wt% could be obtained in the presence of Pt@Hzsm-5 catalysis. Compared with the optimum conditions in the steam-assisted pyrolysis PET reaction without catalysis, the optimum reaction temperature decreased by 50 °C and the yield increased by 12 wt%.

### 3.5. Analysis of the Products of Catalytic Pyrolysis

#### 3.5.1. Analysis of the Solid Products

**Figure 5:**
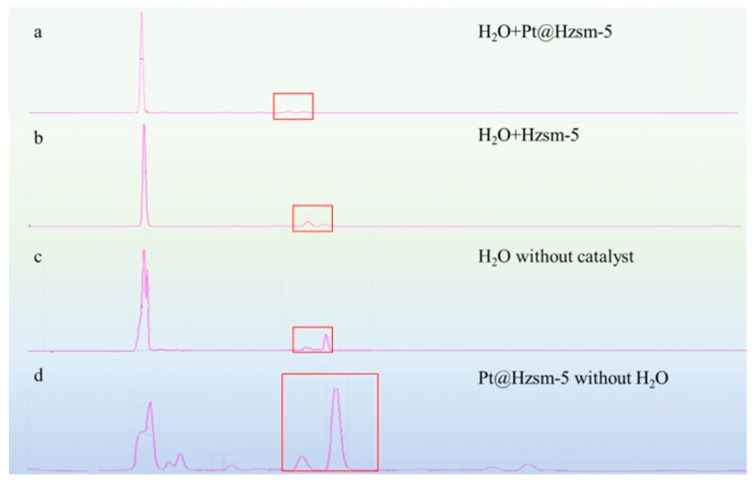
HPLC analysis of the solid products (The main impurity mTPA is in the red suares): (**a**) solid products obtained from the Pt@Hzsm-5 catalytic steam-assisted pyrolysis; (**b**) solid products obtained from the Hzsm-5 catalytic steam-assisted pyrolysis; (**c**) solid products obtained from the steam-assisted pyrolysis without catalyst; (**d**) solid products obtained from the Pt@Hzsm-5 catalytic pyrolysis without water.

As shown in Figure 5, it was noticed that the main solid product of the catalytic steam-assisted pyrolysis of PET is TPA according to the analytical results of the HPLC. Compared to the solid product obtained without catalytic steam-assisted pyrolysis, the purity of the TPA increased from 85.5% to 92.2% with the catalysis of Pt@Hzsm-5. As shown in Figure 5a,c, the improvement in the TPA purity was synchronized with the reduction in the major impurity mTPA. This indicates that the major solid impurity mTPA further cracked to TPA by the action of the catalyst.

At the same time, it was also noticed that the yield of mTPA could also be reduced and the purity of TPA could be improved to more than 90% when only Hzsm-5 was used as a catalyst, as shown in Figure 5b. It is notable that the calculated yields of the TPA obtained in this reaction did not change significantly compared to the uncatalyzed steam-assisted pyrolysis. This indicates that the component that catalyzes the cracking of mTPA to TPA in the reaction process of catalytic steam-assisted pyrolysis of PET is mainly the carrier Hzsm-5. It is also remarkable that in the solid product obtained by catalytic conventional pyrolysis, the purity of the TPA was very low, and a large number of other impurities (mTPA, benzoic acid, etc.) were present. This phenomenon indicates that the catalytic effect of Pt@Hzsm-5 occurs mainly between the water molecule and the PET molecule rather than within the PET molecules.

#### 3.5.2. Analysis of the Gas Products

**Figure 6:**
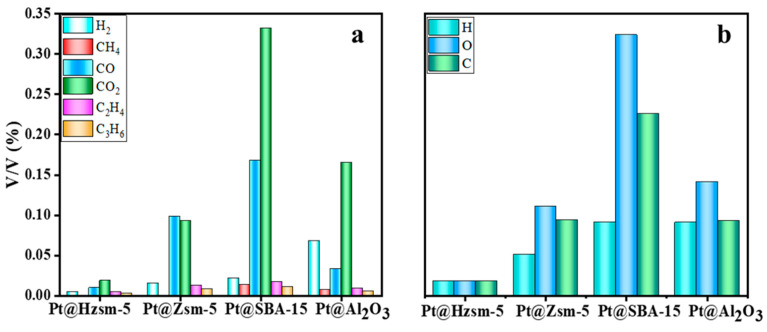
Gas analysis of Pt-based catalysts with different carriers: (**a**) distribution of the gas product components; (**b**) migration of the carbon, hydrogen, and oxygen elements in gases.

In addition to the solid products, the components of the gas-phase products were also analyzed by means of a GC-TCD detector. As shown in Figure 6a, the differences in the gas components corresponding to the Pt-based catalysts with different carriers under the optimized reaction conditions were quite significant. The experimental results show that CO and CO_2_ were still the main gas components in the catalytic steam-assisted pyrolysis reaction. However, the percentage of CO_2_ gas was significantly lower, and the percentage of CO and olefin gas increased in the presence of the Pt@Hzsm-5 and Pt@Zsm-5 catalysts compared to the uncatalyzed steam-assisted pyrolysis reaction35. This not only inhibited the emission of greenhouse gases but also improved the comprehensive utilization value of the gases.

It was also noticed that, contrary to the calculated yield of TPA, the lowest gas concentration was obtained in the reaction with Pt@Hzsm-5 as a catalyst. Since the flow rate of the carrier gas was constant, a lower gas concentration also represents a lower gas release. The carbon, hydrogen, and oxygen elements that escaped as gases in each group of experiments was counted. As shown in Figure 6b, not only the migrated carbon, hydrogen, and oxygen atoms were dramatically reduced by the catalytic effect of the Pt@Hzsm-5, but also the O/C and H/C in the gas were significantly reduced. This was mainly due to the enhanced intermolecular hydrogen transfer efficiency due to the Pt@Hzsm-5, which allows for more hydrogen and oxygen to be immobilized on the TPA in the reaction [22,24].

**Figure 7:**
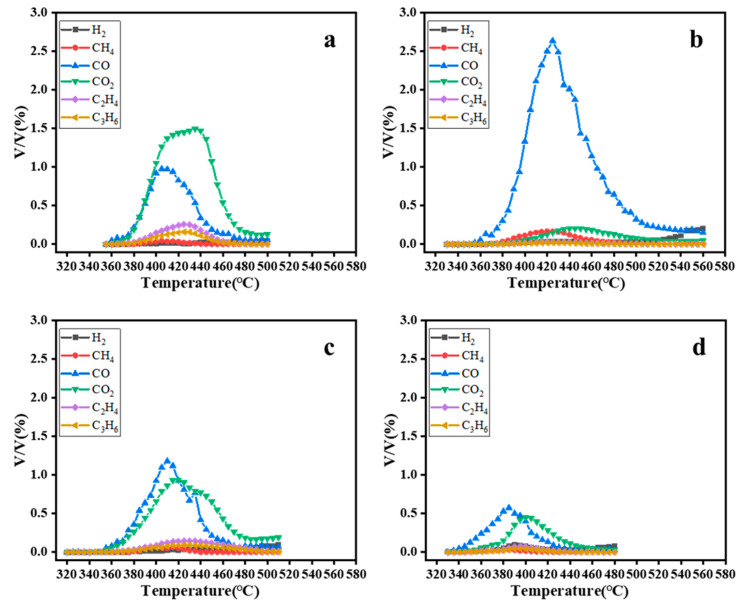
In situ GC analysis of the gas phase product components: (**a**) catalyst-free steam-assisted pyrolysis; (**b**) catalytic conventional pyrolysis (catalyst—Pt@Hzsm-5); (**c**) catalytic steam-assisted pyrolysis (catalyst—Hzsm-5); (**d**) catalytic steam-assisted pyrolysis (catalyst—Pt@Hzsm-5).

In addition, it was also investigated that the gas release with temperature by in-situ GC. As shown in Figure 7, it was carried out that in-situ GC analysis experiments for uncatalyzed steam-assisted pyrolysis, Pt@Hzsm-5 catalyzed conventional pyrolysis, Hzsm-5 catalyzed steam-assisted pyrolysis and Pt@Hzsm-5 catalyzed steam-assisted pyrolysis, respectively. The experimental results show that, unlike other reactions, a large amount of CO can be obtained in the reaction of catalytic conventional pyrolysis of PET, while the amount of CO_2_ released is very low. This is mainly because the catalyst makes the reaction more vigorous, which leads to the release of a large amount of carbon by opening the benzene ring, making the C/O ratio in the reaction system increase rapidly [22]. At the same time, the lack of oxygen brought in by water in the conventional pyrolysis system is also responsible for the further increase of the C/O ratio [22].

Comparing Figure 7a,c, it was found that the concentration of CO was not significantly reduced in the presence of Hzsm-5 only as a catalyst compared to steam-assisted pyrolysis of PET, but the concentration of CO_2_ showed a significant decrease. This is mainly because the monovinyl ester breaks in the presence of Hzsm-5, releasing more carbon atoms and lowering the O/C ratio. Comparing Figure 7c,d, it was found that the peak gas release concentration of both CO_2_ and CO decreased to 50% of the original one when Pt@Hzsm-5 was used as the catalyst. This indicates that the carbon atoms released in the form of gas were substantially reduced, mainly because the excessive cracking of carboxyl and benzene rings is effectively inhibited by the hydrogenation catalytic effect of Pt [22].

In all reactions, C_2_H_4_ was always the earliest gas released, and in Figure 7b,d it was found that the starting temperature of the reaction was 320 °C regardless of the participation of water in the reaction. This indicates that in the reaction system of the steam-assisted pyrolysis of PET, the first reaction to occur was the breakage reaction of vinyl without the participation of water. Moreover, in Figure 7a,d it is noticed that the peak of the gas release was lower than 400 °C in the Pt@Hzsm-5-catalyzed water-assisted pyrolysis reaction, but in the steam-assisted pyrolysis reaction, the peak of the gas release was approximately 450 °C. This phenomenon coincides with the conclusion that the optimal pyrolysis temperature decreased from 450 °C to 400 °C in the presence of catalyst.

### 3.6. Isotope Tracer Experiments

**Figure 8:**
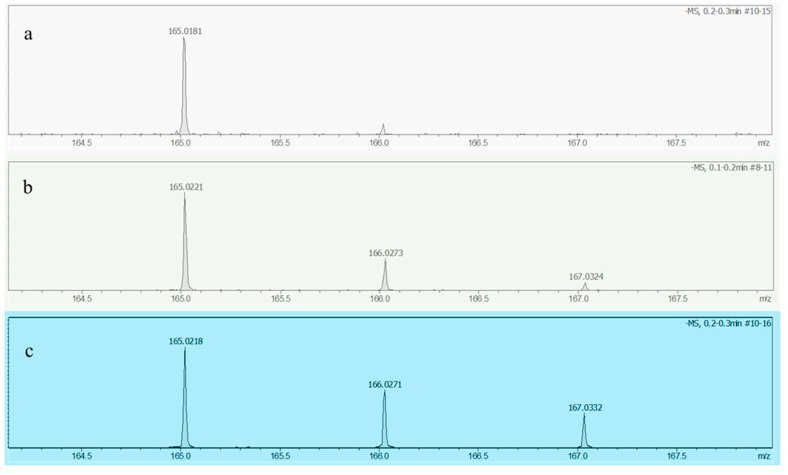
ESI–MS analysis of TPA obtained under different conditions: (**a**) TPA obtained by catalytic steam–assisted (H_2_O) pyrolysis of PET; (**b**) TPA obtained by steam-assisted (D_2_O) pyrolysis of PET without catalyst; (**c**) TPA obtained by catalytic steam-assisted (D_2_O) pyrolysis of PET.

To investigate the reaction mechanism of the catalyst, isotope tracing experiments were designed where D_2_O was used to replace H_2_O in the reaction of steam-assisted pyrolysis of PET. The TPA obtained under three different conditions were analyzed by ESI-MS, as shown in Figure 8.

As shown in Figure 8a, when H_2_O was involved in the reaction, the peak position of the obtained TPA was only 165, but when D_2_O was used instead of H_2_O, the peak positions of TPA were 166 and 167 in addition to 165. This is because when D_2_O is involved in the reaction, some deuterium atoms replace the hydrogen atoms in PET to appear on the TPA molecule [22]. Among them, position 166 corresponds to mono-deuterated TPA, and position 167 corresponds to di-deuterated TPA. Meanwhile, combined with previous studies [22], the three TPA molecules correspond to three reaction pathways, namely, thermal cracking, hydroxyl attack, and hydrogen transfer.

As shown in Figure 8b,c, while D_2_O is involved in the reaction, three molecules of TPA, mono-deuterated TPA, and di-deuterated TPA appeared simultaneously with or without the action of the catalyst. However, the intensity of the peaks at positions 166 and 167 increased significantly under the catalytic effect of Pt@Hzsm-5. This phenomenon indicates that the percentage of mono- and di-deuterated TPA molecules in the product increased. This implies that Pt@Hzsm-5 can effectively catalyze the intermolecular hydrogen transfer reaction, allowing for more hydrogen in the water to complement the hydrogen that escaped during the PET pyrolysis, thus achieving the goal of inhibiting excessive cracking.

**Figure 9:**
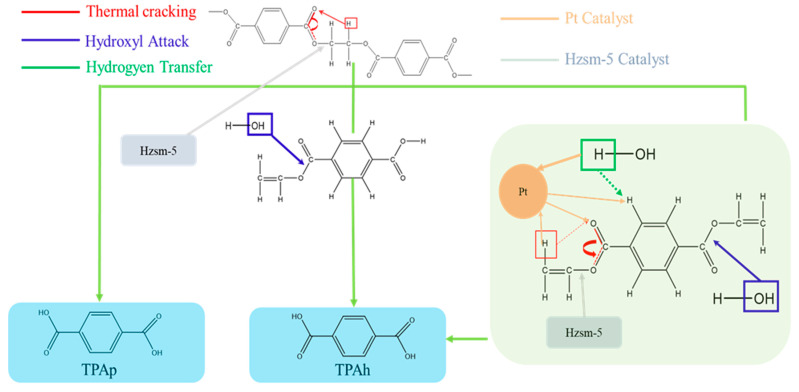
Reaction pathway of the Pt@Hzsm-5-catalyzed steam-assisted pyrolysis of PET.

As shown in Figure 9, the generated TPA can be divided into two categories according to whether or not hydrogen transfer occurs with water molecules: TPA derived entirely from thermal cracking (TPAp) and TPA derived with the participation of water in the reaction (TPAh). The corresponding three reaction pathways are thermal cracking, hydroxyl attack, and hydrogen transfer. In addition, the role of Pt@Hzsm-5 in the reaction process of catalytic steam-assisted pyrolysis of PET could be divided into two parts: catalytic hydrogenation and catalytic cracking, which are performed with Pt and Hzsm-5, respectively.

As shown in Figure 9, the main reaction path of Hzsm-5 is to catalyze the thermal cracking reaction of mTPA, which reduces impurities to improve the purity of TPA and increases the yield of olefins. The main reaction pathway catalyzed by Pt is the hydrogen transfer between water molecules and PET molecules, especially with benzene ring hydrogen molecules [22]. Thus, the purpose of inhibiting excessive cracking and improving the calculated yield of TPA was achieved.

### 3.7. Characterization of the Catalyst

**Figure 10:**
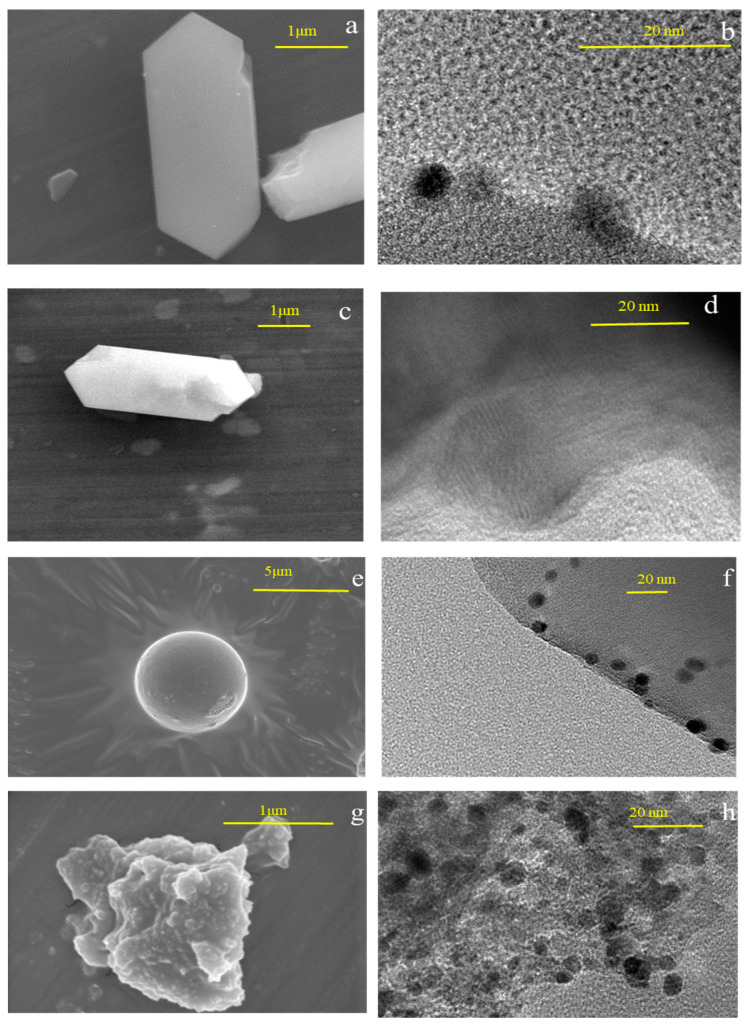
SEM/TEM analysis of the catalyst: (**a**) SEM analysis of Pt@Zsm-5; (**b**) TEM analysis of Pt@Zsm-5; (**c**) SEM analysis of Pt@Hzsm-5; (**d**) TEM analysis of Pt@Hzsm-5; (**e**) SEM analysis of Pt@SBA-15; (**f**) TEM analysis of Pt@SBA-15; (**g**) SEM analysis of Pt@Al_2_O_3_; (**h**) TEM analysis of Pt@Al_2_O_3_.

To investigate the morphological structure of the Pt catalysts, SEM and TEM analyses were performed on the catalysts used, and the results are shown in Figure 10. It was noticed that both Hzsm-5 and Zsm-5 are regular hexagonal structures. However, the size of the loaded metal clusters on Hzsm-5 is smaller, less than 10 nm in diameter, while the diameter of the loaded metal clusters on Zsm-5 is above 20 nm. The SBA-15 observed under SEM is a spherical structure, but with a larger size, approximately 5 μm in diameter, and the diameters of the loaded metal clusters are between 10 and 20 nm. The morphological structure of the directly crushed Al_2_O_3_ showed an irregular lamellar structure with a particle diameter of approximately 1 μm. The density of the loaded metal clusters on this carrier was relatively higher, with an average diameter of more than 10 nm.

### 3.8. Catalyst Recycling Experiments

**Figure 11:**
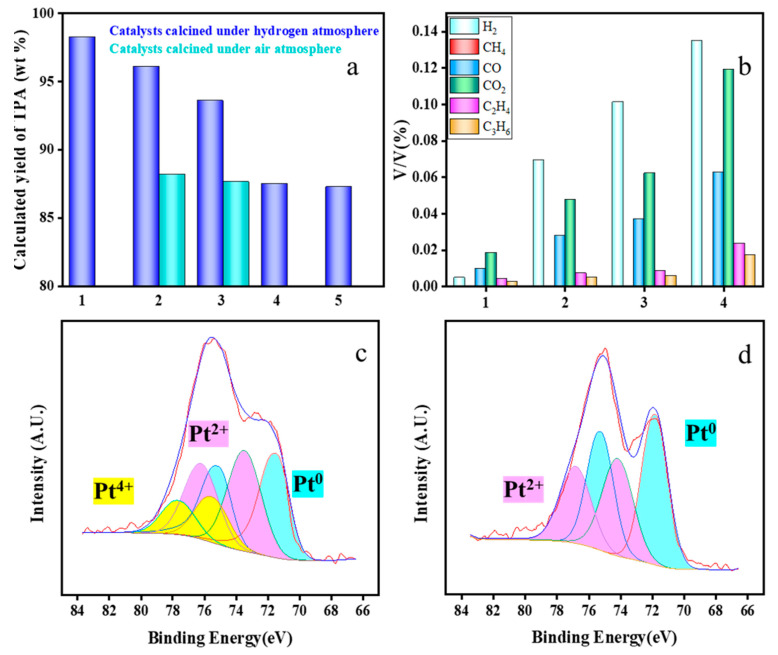
Product distribution in the catalyst recycling experiments: (**a**) variation of the calculated yield of TPA; (**b**) variations of the gas components; (**c**) XPS analysis of the catalysts after air calcination; (**d**) XPS analysis of the catalysts after hydrogen calcination. (The yellow area is Pt^4+^, the purple area is Pt^2+^ and the blue area is Pt^0^.)

As a nonhomogeneous cracking catalyst, the problem of carbon accumulation on the surface of Pt@Hzsm-5 is unavoidable. It was removed that the surface carbon accumulation by calcination at 750 °C and verified the catalytic activity of the nascent catalyst. As shown in Figure 10a, when calcination was performed in air, the activity of the obtained catalysts was low and similar to the catalytic effect of Hzsm-5. However, when the catalyst was calcined in hydrogen, the catalytic activity of the catalyst was recovered to some extent and could be recycled three times, with a decrease in the yield occurring on the fourth occasion. Correspondingly, as shown in Figure 11b, the gas concentration increased gradually with the number of cycles.

The XPS characterization of the catalysts calcined in both atmospheres were performed, and the results are shown in Figure 11c,d. The more highly valent state of platinum was clearly detrimental for catalysis, while calcination in a hydrogen atmosphere allowed for the conversion of the highly valent state of platinum to the less valent and singlet states. However, as the number of cycles increased, the Pt catalyst after multiple calcinations still inevitably deactivated, which may be mainly caused by metal agglomeration and migration at high temperatures.

## 4. Conclusions

The catalytic steam-assisted pyrolysis of PET is an effective method to improve the calculated yield and purity of TPA. In this reaction system, the metal–acid catalyst possessed good catalytic activity. Among them, the catalytic activities of the carriers were in the order of Hzsm-5 > Zsm-5 > Al_2_O_3_ > SBA-15, and the catalytic activity of Pt was better than that of Pd. The optimum temperature of the steam-assisted pyrolysis of PET decreased to 400 °C with the Pt@Hzsm-5 catalyst, the highest calculated yield of TPA reached 98.23 wt% with a 92.2% purity, and the minimum excess vapor coefficient (α) required for the reaction decreased to 110. The Pt@Hzsm-5 catalyst could be recycled three times by calcination for carbon removal and hydrogen reduction.

Through analysis of the products, in situ GC experiments, and isotope tracing experiments, the reaction mechanism of Pt and Hzsm-5 synergistically catalyzed PET vapor-assisted pyrolysis was clarified. It was determined that the catalytic effect of Pt mainly targets the intermolecular hydrogen transfer reaction between water molecules and PET, while Hzsm-5 mainly catalyzes the reaction of the continued cracking of mTPA to TPA. This study shows that metal–acid catalysts such as Pt@Hzsm-5 can play a significant role in the reaction of steam-assisted pyrolysis of PET. It lays the foundation for the further exploration of other kinds of catalysts and eventually the industrialization of TPA preparation by PET pyrolysis.

## Data Availability

Not applicable.

## Supplementary Materials

The following supporting information can be downloaded at: https://www.mdpi.com/article/10.3390/ma16062362/s1, Figure S1: Schematic diagram of the experimental facility and process pathway. 1. N2; 2. Water; 3. Flow meter; 4. Micro-pump; 5. Preheater; 6. Metal reaction tube; 7. Quartz reaction tube; 8. Quartz basket; 9. Temperature controller; 10. Filter for collecting powder; 11. Condenser; 12. Circulating pump; 13. Liquid collecting tank; 14. Filter; 15. Micro-GC. Figure S2: Characterization of carriers (a) BET (b) NH_3_-TPD
