# Supplementary material for: Catalytic Steam-Assisted Pyrolysis of PET for the Upgrading of TPA
Source: Materials (Basel). 2023 Mar 15;16(6):2362. doi: 10.3390/ma16062362 (PMC10052107; doi:10.3390/ma16062362)
Supplement: Supplementary file 1 [file materials-16-02362-s001.zip › materials-2243345-supplementary.pdf]

Supplementary Materials

Supplementary Note 1: Experimental conditions and equipment

The commercial PET used in these experiments were purchased from DuPont in the form of chunks of about 5mm × 4 mm × 3mm size. These chunks were frozen to -196°C by liquid nitrogen, and then grinded and sieved to obtain the fraction below 80 mesh. Before PET was pyrolysised by a fixed-bed reactor, the raw materials were dried at 105°C for 8h.

The water (H_2O) used during this research was deionized water; the D_2O was produced by Aladdin company with a purity of 99.995%; the methanol and DMSO purchased from Aladdin are all chromatographically pure.

In order to explore the law of PET pyrolysis, a set of dedicated fixed bed reactor was built (as shown in S. Figure. 1). The reaction system included a nitrogen flow valve, a micropump, a preheating reactor, a vertical tube reactor, a set of condensing equipment, a bag filter for collecting solids, and an in-situ GC-TCD gas detector. The reactor is made of 310S (ASTM) steel pipe (outer diameter is 38mm, wall thickness is 3mm, length is 600mm), and the lining is quartz tube (outer diameter is 30mm, wall thickness is 2mm, length is 600mm).

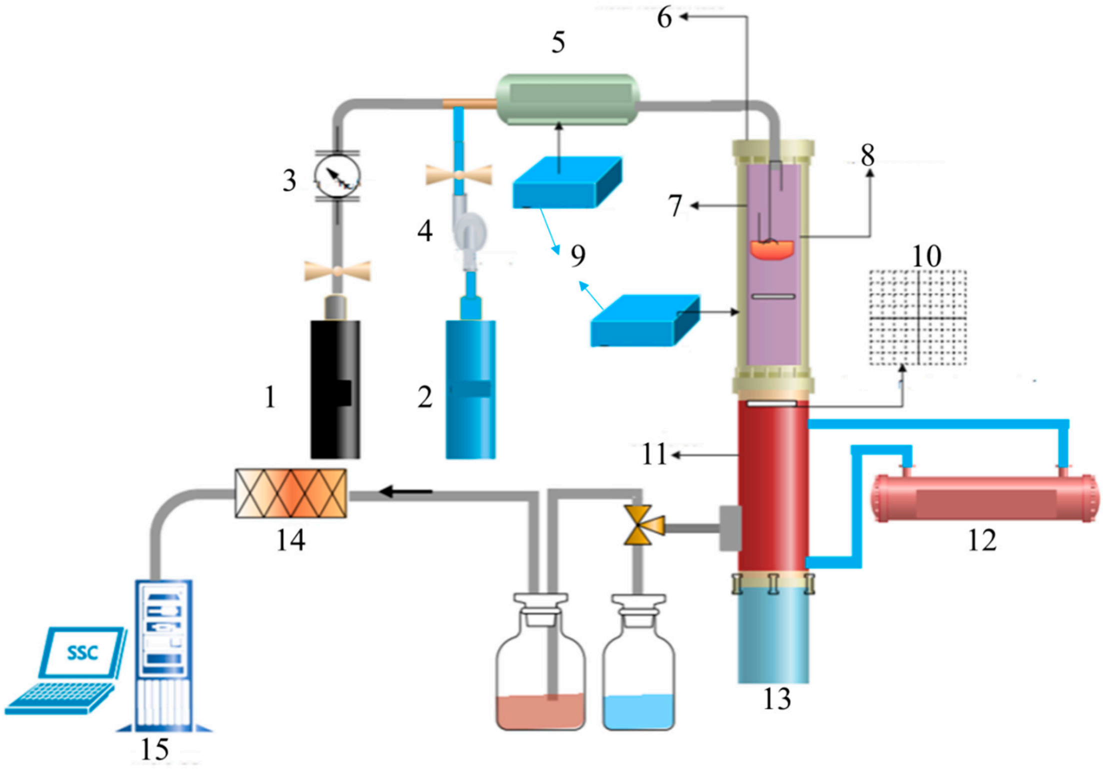

Supplementary Figure S1. Schematic diagram of the experimental facility and process pathway

1. N_2; 2. Water; 3. Flow meter; 4. Micro-pump; 5. Preheater; 6. Metal reaction tube; 7. Quartz reaction tube; 8. Quartz basket; 9. Temperature controller; 10. Filter for collecting powder; 11. Condenser; 12. Circulating pump; 13. Liquid collecting tank; 14. Filter; 15. Micro-GC

In the above experiment, we found that water inhibited the formation of carbon black, but when it exceeded a certain range, adding more water had no significant effect on inhibiting the formation of carbon black. In order to quantify the effect of water on the pyrolysis of PET, we defined the excess steam coefficient (α) to explore the change of TPA:

$$\alpha = m1/m2 \tag{1}$$

Among them, m1 and m2 are respectively the total amount of water consumed within the temperature range where the reaction could occur (T>320° C) and the amount required for complete hydrolysis of the PET participating in the reaction.

When the quality of PET was determined, the value of m2 was fixed, but the value of m1 depended on the combined effect of the final pyrolysis temperature (T), residence time (t), water flow rate (F), and heating rate (H):

$$m_1 = [(T - 320)/H+t]*F \tag{2}$$

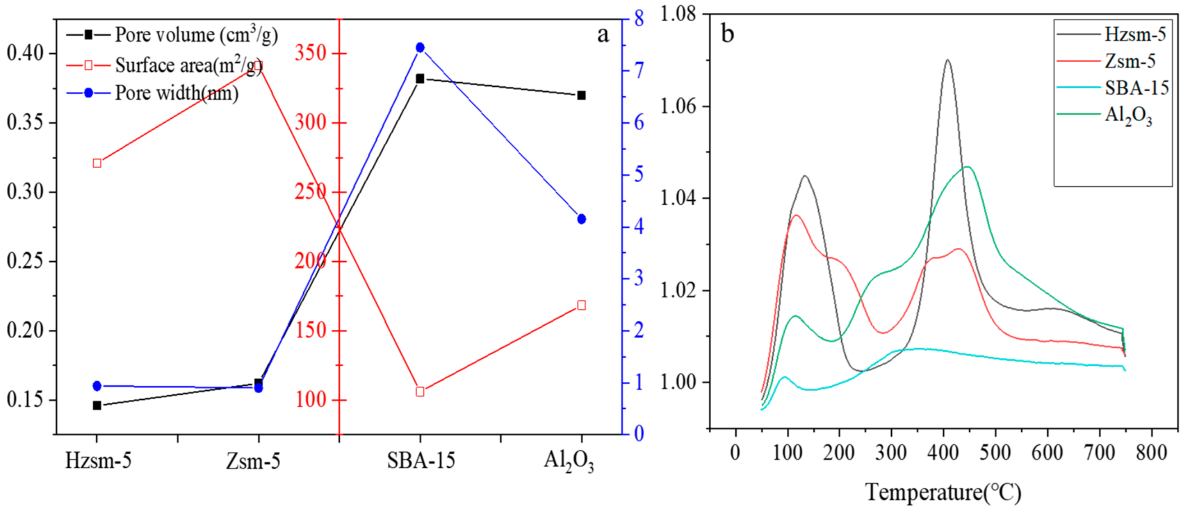

Supplementary Figure S2. Characterization of carriers (a) BET (b) NH_3-TPD
